# Animal bite anaphylaxis, rarely diagnosed but underappreciated 

**DOI:** 10.5414/ALX02421E

**Published:** 2023-08-22

**Authors:** Gregg M. Stave

**Affiliations:** Division of Occupational and Environmental Medicine, Duke University, Durham, NC, USA

**Keywords:** animal allergy, pet allergy, laboratory animal allergy, occupational allergy, occupational anaphylaxis

## Abstract

Background: Animal allergies are common, with reactions ranging from rhinoconjunctivitis from respiratory exposure to anaphylaxis, usually from animal bites. Since animal bites are also common, this raises the question of how often anaphylaxis occurs following a bite. Materials and methods: A PubMed, Google, and Google Scholar literature review was performed using keywords such as animal bite anaphylaxis. An inquiry was made to the Anaphylaxis Registry ANAPHYLAXIE.net to see if additional cases were contained in the registry. Results: Approximately 40 cases of animal bite anaphylaxis are described in the literature, mostly from rodent bites (mice, rats, hamsters, and guinea pigs). A survey of laboratory animal bite anaphylaxis in the U.S. identified previously unreported cases, suggesting that most cases are not reported. Conclusion: Anaphylaxis from animal bites is rarely reported, but occurs more frequently than suggested by case reports and should be considered in a symptomatic patient following a bite.

## Introduction 

Pet ownership is common with surveys indicating that globally more than one half of people own pets, with dogs owned by one in three [1]. Cats are next most common, followed by a variety of other animals. Unfortunately, animal bites from pets are also common. While there are no global estimates, data suggests that in the U.S. ~ 4.5 million people are bitten by dogs and 400,000 by cats each year [2]. There is also a large laboratory animal research workforce, which is also at risk for animal bites. Animal bites are of concern because in addition to trauma, they can become infected or transfer infections such as rabies or tetanus. 

Allergies to animals are also common, with an estimated worldwide prevalence of 10 – 20% [3]. For laboratory animal workers, the prevalence of allergy has been estimated to be between 11 and 44% [4]. Animal allergy usually manifests as rhinoconjunctivitis, but may result in dermal symptoms, asthma, sinusitis, or anaphylaxis. 

Anaphylaxis is a severe systemic allergic reaction, usually initiated by immunoglobulin E (IgE) triggering release of mediators from basophils and mast cells. It is unclear what triggers the pathway that leads to a systemic anaphylactic reaction instead of a simple local allergic reaction [5]. 

For animal allergies, the most common instigating IgE molecules have been elucidated. Many of these allergens are members of the lipocalin family [4, 6]. These molecules have a common tertiary structure with substantial sequence homology, and an internal binding pocket carries small hydrophobic molecules such as retinol, steroids, odorants, and pheromones [6]. Animal allergen lipocalins include those of mouse (Mus m 1), rat (Rat n 1), guinea pig (Cav p 1, Cav p 2, Cav p 3, Cav p 6), hamster (Mes a 1, Phod s 1), rabbit (Ory c 1, Ory c 2, Ory c 4), dog (Can f 1, Can f 2, Can f 4, Can f 6), cat (Fel d 4, Fel d 7), and horse (Equ c 1, Equ c 2) [6]. However not all animal allergens are lipocalins. For example, the primary cat allergen, Fel d 1 is a secretoglobin [7]. A major dog allergen, Can f 5, is a member of the kallikrein family [8]. These allergens can be found in saliva, dander, hair, and urine, as well as places where animals have been. 

Depending on the definitions used, study methodology, and geographical areas, the global incidence of anaphylaxis ranges from 50 to 112 episodes per 100,000 person-years with an estimated lifetime prevalence of 0.3 – 5.1% [9]. While most cases are associated with medications, many are related to food, insect stings, non-rubber latex, chemicals, and bites from mammals [10]. 

A study published in 2017 on animal bite anaphylaxis in the research laboratory setting suggested that anaphylaxis from animal bites occurred more frequently than was indicated by prior published case studies [11]. This led to the question of how frequently anaphylaxis occurs following bites from animals – is it rare or underappreciated? 

## Materials and methods 

To identify cases of anaphylaxis associated with animal bites, a search for English language publications was conducted using PubMed, Google, and Google Scholar, using keywords such as animal bite anaphylaxis, laboratory animal bite anaphylaxis, and pet bite anaphylaxis. An iterative search was performed for additional cases identified in references. In addition, an inquiry was made to the Anaphylaxis Registry ANAPHYLAXIE.net, a voluntary registry with participation from physicians in Germany, Austria, Switzerland, other European countries, and Brazil, to determine if any cases had been reported to the registry. 

## Results 

### Cases of anaphylaxis involving pets in the literature 

Anaphylaxis following animal bites from pets has been reported for mice [12], hamsters [12, 13, 14, 15, 16], a gerbil [18], and a cat [19]. There are several reports of anaphylaxis from the bite of a slow loris [20, 21, 22, 23], which is sometimes kept as a pet. This may be due to the fact that the venom has a sequence that is similar to the Fel d 1 cat allergen [24]. There are also reports of anaphylaxis following inhalation exposure to a cat [25] and a rabbit [26] with no history of a bite. 

Of these case reports, 7 involved hamsters [12, 13, 14, 15, 16], including 5 from dwarf hamster bites [14, 15, 17]. Testing revealed a positive response to a variety of allergens: proteins in hamster saliva [12], IgE against *Dermatophagoides pteronyssinus* (Der p) and positive skin test reactions to hamster saliva [13], IgE-mediated reaction to a novel 21 kD protein found in the dwarf hamster saliva which had some cross-reactivity with Der p [15], IgE to hamster epithelium [14], specific IgE antibodies against dwarf Djungarian hamster (*Phodopus sungorus*) epithelium [14], and a new allergenic protein belonging to the lipocalin family that was identified as a possible cause of a reaction that lacked cross-reactivity with allergens from other rodents [17]. 

Additional reports involved mouse [12], gerbil [18], and cat [19] bites resulting in anaphylaxis. For the mouse bite, skin prick tests showed sensitization to dog hair, cat hair, horsehair, and mouse epithelia, and testing also revealed IgE antibodies against egg and mouse epithelia [12]. For the gerbil bite, IgE antibodies were identified against mice, cats, hamsters, and rats, and a skin provocation test with soaked hairs from the gerbil was positive [18]. For the cat bite, testing revealed IgE antibody to cat dander [19]. 

### Cases of anaphylaxis from laboratory animal bites 

More cases of anaphylaxis from laboratory animals are described in the literature than for pet bites. Mice and rats are the most commonly used research animals. More than 30 cases have been reported involving bites to the fingers and hands from rodents, including those identified through the U.S. national survey [11]. In these cases, the workers ranged in age from 23 to 55. Eight had no history of any form of allergy or asthma [11, 27, 28]. Among those with a prior history of respiratory and/or dermal allergy, the interval between initial allergy symptoms and the episode of anaphylaxis ranged from less than 1 year to 12 years [11]. Four had histories of previous animal bites that did not result in an anaphylactic response [28, 29, 30, 31]. All workers recovered, although 1 required cardiopulmonary resuscitation and intubation [32]. Eleven workers had subsequent allergen testing with laboratory animal extracts (skin prick test and/or IgE), and all had positive test results [11, 27, 28, 29, 30, 31]. 

### Anaphylaxis registry 

In 2006, the anaphylaxis registry ANAPHYLAXIE.net began collecting voluntary reports from physicians in German-speaking countries and has since expanded to include other European countries and Brazil [33]. As of March 31, 2023, the registry had over 12,000 cases and no reports of anaphylaxis from pet bites [34]. 

## Discussion 

While bites from pets are common, a review of the literature and an anaphylaxis registry identified only a small number of case reports of anaphylaxis from pet bites. Most of these cases involved rodents, although there is 1 case involving a cat [19] and 4 from the bite of a slow loris [20, 21, 22, 23]. A larger number of cases associated with laboratory animal bites are described in the literature. Remarkably, there were no published reports of anaphylaxis following dog bites. 

The low number of reports of anaphylaxis following animal bites suggests that this may be a rare phenomenon. However, case reports likely underestimate the incidence of anaphylaxis for several reasons. Case reports are usually published from academic centers, which care for a minority of patients. Cases get reported because there is something novel, so case reports are seldom written when something is common. For example, 3 of the published cases associated with bites from pets involved unexpected allergens: 2 with Der [14], associated with house dust mites, and 1 with a novel allergen [15]. 

For anaphylaxis following laboratory animal bites, case reports underestimated the incidence [11]. There were 9 case reports in the literature prior to a U.S. national survey of animal research facilities conducted in 2016. That survey identified 23 additional cases of anaphylaxis following bites. While the majority of the 198 research organizations that responded reported no cases, 15 organizations reported that workers had experienced anaphylaxis following an animal bite. It is notable that 3 organizations had 2 cases, 1 had 3 cases, and 2 had 4 cases [11]. An organization experiencing several cases wouldn’t consider it rare [35]. As illustrated by anecdotal experience, not all cases are reported to employee health departments, such as a not previously reported mouse bite to a graduate student resulting in anaphylaxis in a university setting where the incident was initially handled through student health [36]. 

If case reports underestimate the incidence, then how commonly do anaphylactic reactions occur following bites? Klotz et al. [37] write that “Emergency Physicians frequently see patients who complain of an allergic reaction to an animal bite”. However, data on incidence is not provided. 

The lack of cases reported to the anaphylaxis registry suggests that the problem is uncommon. The registry is a valuable resource that collects voluntary reports from physicians from many countries in Europe and Brazil. However, physicians may be selective in their reporting. In 2014, Worm et al. [38] published an analysis of more than 4,000 cases in the registry from Germany, Austria, and Switzerland. The most common triggers of anaphylaxis were wasp and bee venom, legumes, animal proteins in food, and analgesic drugs. Looking at these cases using a severity grading system modified from the classifications proposed by Ring and Messner [39], it is notable that fewer than 10 cases were classified in the least severe category [38]. If the full spectrum of anaphylaxis was represented, it is likely that a higher percentage of all cases would fall into the least severe category, which suggests that physicians may underreport these cases. 

Similarly, a retrospective review of 516 patients with anaphylaxis seen in a tertiary adult allergy clinic in Turkey did not include any cases due to animal bites [40]. In this analysis, only 4.9% of patients presented with the lowest level of severity, an acute onset of illness with cutaneous or mucosal involvement associated with respiratory compromise or cardiovascular compromise. As with the registry, while it is possible that less severe symptoms are uncommon, it probably indicates that physicians are less likely to refer patients with less severe symptoms to a tertiary care clinic. 

The spectrum of severity of anaphylaxis following animal bites would be expected to be similar to anaphylaxis with other causes. So, even if the registry and case series may lack less severe cases, the absence of cases suggests that anaphylaxis after pet bites is uncommon. 

Another avenue to explore when seeking to understand the incidence of anaphylaxis from pet bites is to look at anaphylaxis associated with immunotherapy. While not a perfect comparator, subcutaneous allergen immunotherapy (SCIT) has some similarity to exposure that occurs during an animal bite. The biggest differences are that the quantity and location of allergen exposure are precisely defined with SCIT, unlike a bite, which also has the potential for a mix of allergens. The rates of anaphylaxis associated with SCIT was estimated to be ~ 0.2% per injection [41]. However, there are no studies that break down the rates of reaction by type of allergen. 

Considering all available information, the data does not permit the calculation or estimation of the incidence of animal bite anaphylaxis. While some data, such as the lack of cases in the anaphylaxis registry, suggest that these events are rare, reports that some research centers have seen 3 or 4 cases resulting from laboratory animal bites suggests that it may be more common. If so, it may also be underappreciated. 

## Conclusion 

Anaphylaxis from animal bites is rarely reported with only approximately 40 cases described in the English language literature. However, anaphylaxis occurs more frequently than suggested by published case reports and should be considered in a symptomatic patient following a bite. 

## Funding 

None. 

## Conflict of interest 

None. 
